# Mechanisms and factors affecting the removal of minocycline from aqueous solutions using graphene-modified resorcinol formaldehyde aerogels

**DOI:** 10.1038/s41598-023-50125-0

**Published:** 2023-12-20

**Authors:** Amirhosein Yazdanbakhsh, Alireza Behzadi, Armaghan Moghaddam, Iman Salahshoori, Hossein Ali Khonakdar

**Affiliations:** 1 Department of Polymer Processing, Iran Polymer and Petrochemical Institute, Tehran, Iran; 2 Department of Polymer Science, Iran Polymer and Petrochemical Institute, Tehran, Iran

**Keywords:** Chemistry, Engineering, Materials science

## Abstract

In recent years, concerns about the presence of pharmaceutical compounds in wastewater have increased. Various types of residues of tetracycline family antibiotic compounds, which are widely used, are found in environmental waters in relatively low and persistent concentrations, adversely affecting human health and the environment. In this study, a resorcinol formaldehyde (RF) aerogel was prepared using the sol–gel method at resorcinol/catalyst ratio of 400 and resorcinol/water ratio of 2 and drying at ambient pressure for removing antibiotics like minocycline. Next, RF aerogel was modified with graphene and to increase the specific surface area and porosity of the modified sample and to form the graphene plates without compromising the interconnected porous three-dimensional structure of the aerogel. Also, the pores were designed according to the size of the minocycline particles on the meso- and macro-scale, which bestowed the modified sample the ability to remove a significant amount of the minocycline antibiotic from the aqueous solution. The removal percentage of the antibiotic obtained by UV–vis spectroscopy. Ultimately, the performance of prepared aerogels was investigated under various conditions, including adsorbent doses (4–10 mg), solution pHs (2–12), contact times of the adsorbent with the adsorbate (3–24 h), and initial concentration of antibiotic (40–100 mg/l). The results from the BET test demonstrated that the surface area of the resorcinol formaldehyde aerogel sample, which included 1 wt% graphene (RF-G1), exhibited an augmentation in comparison to the surface area of the pure aerogel. Additionally, it was noted that the removal percentage of minocycline antibiotic for both the unmodified and altered samples was 71.6% and 92.1% at the optimal pH values of 4 and 6, respectively. The adsorption capacity of pure and modified aerogel for the minocycline antibiotic was 358 and 460.5 mg/g, respectively. The adsorption data for the modified aerogel was studied by the pseudo-second-order model and the results obtained from the samples for antibiotic adsorption with this model revealed a favorable fit, which indicated that the chemical adsorption in the rapid adsorption of the antibiotic by the modified aerogel had occurred.

## Introduction

In recent decades, one of the biggest environmental issues is the risk associated with the pollution in drinking water caused by organic compounds^1^. Among all organic compounds that have found their way into water, antibiotics may be posing one of the biggest threats^2^. They can enter the water resources as a cause of mismanagement in the pharmaceutical industry, hospital wastewater, and human and animal excrement^3^. While antibiotics are now the most widely used method of treating infections and the community's health relies on them, according to several studies, their overuse has been the primary cause of the rise in antibiotic-resistant bacteria in recent years^4^. When treating infections, bacterial resistance can be transferred to the genetic makeup of the bacteria in cases where the concentration of the antibiotic is lower than the minimum inhibitory concentration; since even in low contents, they can cause accumulative resistance that is a serious threat to both the patient and the healthcare system^5^. Nevertheless, the complete effects of minor antibiotic doses or low concentrations on the human body remain not entirely comprehended^5,6^. On the other hand, antibiotics in drinking water can potentially enter the body, increase the concentration of these substances in the tissues, and trigger a variety of responses^7^. Investigations have revealed that the chronic effects of antibiotics outweigh their acute impacts^8^. Hence, an accumulative antibiotic uptake in the body due to its presence in water should be prevented. In addition, antibiotics might impact the bacterial colonies in the wastewater system, and the activity of bacteria is hindered when antibiotics are present in wastewater treatment systems, substantially impairing the procedure of treatment and breakdown of organic matter^9,10^. Should antibiotics remain present in wastewater due to ineffective treatment, there is a possibility that they will seep into surface water reservoirs, impacting various organisms within the food chain^11^. Algae are extremely susceptible to several antibiotics, and the food chain starts with them. Therefore, even a slight decrease in their numbers might affect the equilibrium of the food chain^12^. In terms of nanograms to micrograms per liter (ng/L -µg/L), the content of residual (unrefined) antibiotics in water is quite low. Yet, their accumulation in animals, plants, and poultry can put the whole ecosystem at risk^13^. Thus, several initiatives and experiments are being made to determine the best way to eliminate them from water supplies.

Minocycline antibiotics are among the most extensively used and prevalent tetracycline antibiotics. These antibiotics are frequently employed to treat both humans and animals. Numerous bacterial illnesses of the intestines, skin, urinary tract, respiratory tract, genitalia, lymph nodes, and other body parts are treated with them. They are referred to as universal antibiotics. These antibiotics have a structure made up of four aromatic rings and three distinct substituents^14,15^. The majority of removal techniques for the tetracycline family use biological and physicochemical treatment approaches. Nevertheless, since sufficient design and operational expertise has not been obtained yet, most biological approaches for treating tetracycline-containing wastewater are still in the experimental stages. Besides, photocatalytic oxidation, coagulation, adsorption, and ozone oxidation are some of the current popular physio-chemical approaches for treating tetracyclines effluent^16,17^. While the coagulation technology has the advantages of flexible operation time, simple equipment, and effective performance in refining, unfortunately, the problem with managing chemical sludge has constrained coagulation technology^18^. On the other hand, since ozone technology has been regarded as an effective approach thanks to its rapid reaction time and potent oxidation activity, engineers face other problems when using this method due to the high consumption of hazardous chemicals and ozone formation^19^. Procedures for photocatalytic oxidation have quick response times, are inexpensive, and produce no secondary pollutants^20^. Nevertheless, most of them are still in the research and theoretical stages because of their limited application scope, inability to be recycled, challenging reactor design, high energy usage, and so forth. However, adsorption technology is superior to the previously described techniques for removing tetracyclines since it is straightforward, easy to use, highly effective, relatively inexpensive, and does not result in toxic byproducts^21^.

Most commonly used adsorbents to remove minocycline antibiotic from aqueous solutions, include biochars (BCs)^22^, chitosan^23^, activated carbon^24^, metal organic frameworks (MOFs)^25^, biomass^26^, and mesoporous/clay materials^27^. A suitable adsorbent for minocycline antibiotic removal is an adsorbent that has a high specific surface area, a porous structure with high porosity, tailored pore size range and high active sites.

Biomass is one of the most abundant and economical organic raw materials that can be recycled on earth, and can be obtained from wood, forest products, sludge, fertilizer, agricultural waste and organic waste^28^. Biochar with different physio-chemical properties and different adsorption properties is easily produced by biomass waste^29^. Biochar as adsorbent has a porous structure, high specific surface area and hydrophobic, which makes it a suitable adsorbent for removing various pollutants from wastewater^30^. Biochars are made from algae, wood chips, palm leaves, rice straw and pyrolysis of carbonaceous materials (such as wood and paper)^31,32^. Chitosan is a polysaccharide which is widely utilized in many fields due to having interesting properties such as sustainability, biocompatibility, and multiple active groups^33^. While chitosan has a high content of free amino and hydroxyl groups on the backbone, that can form hydrogen bonds and help the removal of antibiotics, chitosan has disadvantages such as low chemical stability, solubility in acidic conditions, and weak mechanical properties^34^. MOFs are novel materials that are formed through the self-assembly of organic linkers and metal ions^35^. Since they have high specific surface areas, they have been reported in studies for antibiotic removal^36^. Nonetheless, using MOFs in aqueous solutions is not feasible as their structure collapses in water^37^.

According to the properties expected from removal of antibiotics, aerogels are suitable candidates that can have the ideal adsorbent properties and remove minocycline antibiotic to a significant level. Aerogels are classified into three categories: organic, inorganic and hybrid^38^. One of the most common organic aerogels is resorcinol formaldehyde aerogel. In the synthesis of resorcinol formaldehyde aerogel, two parameters including resorcinol/catalyst (R/C) and resorcinol/water (R/W) are very important^39^. In previous studies, under the same conditions, resorcinol formaldehyde aerogel was synthesized at R/C = 50–1000 and R/W = 2, and RF aerogel had the highest specific surface at R/C = 400. R/C lower than 50 and higher than 1000 was not investigated because in this R/C no gel is formed at all^40^.

Aerogels are open-cell, porous materials with low density and a high percentage (over 98%) of internal voids^41^. They have unique characteristics, such as low heat conductivity and large specific surface area. They are suitable for specific constructional, electrical, medical, and environmental applications because of their unique properties^42,43^. A novel class of integrated aerogels may now be synthesized thanks to organic precursors that can create stable covalently linked (C–C) organic polymers^44^. Resorcinol formaldehyde (RF), recognized as a prominent organic aerogel, is created by sequentially polymerizing resorcinol and formaldehyde with a catalyst in an aqueous environment, typically alkaline and occasionally acidic^40^. To craft the aerogel, diverse techniques can be employed to eliminate the medium from the wet gel resulting from the sol–gel reaction. These methods encompass freeze-drying, supercritical drying, and ambient pressure drying^45^. Within those, drying in ambient pressure does not require high energy consumption, high pressures, autoclaves, or extractors; it is inexpensive and can be used to mass-produce aerogels^46^. Unlike carbon-based aerogels, which must be pyrolyzed to remove antibiotics, RF aerogels may be altered while being synthesized to do so. Further, they may be produced at low temperatures with little or no excessive energy consumption^47^.

The number of studies on the application of aerogels for antibiotic removal from water has increased recently as a result of the special benefits and traits of aerogels in this field^48–50^. Unlike techniques such as membrane processes and biological procedures that do not destroy antibiotics and transfer them from one phase to another, aerogels eliminate antibiotics at high rates by adsorption^51^. Aerogels can be modified by nanomaterials with high surface areas that can form interactions with the adsorbate such as graphene to increase their efficiency of antibiotic removal^52^. While graphene-based aerogels are widely studied for their adsorption capacities, they have shown better properties and performance when combined with polymers^53,54^. Hence, in this study, RF aerogel was prepared by the sol–gel method and ambient-pressure drying for the removal of minocycline antibiotic; also, the properties along with the effect of adsorption conditions on the process efficiency were investigated.

In our previous works^40,55^, the removal of tetracycline antibiotics by RF aerogel and their modification with 1 wt% meta-phenylenediamine and 1,3 wt% amine-functionalized graphene oxide (GO) have been reported. In this study, the removal of minocycline antibiotic was enhanced by modification of RF aerogel with 1 wt% graphene. Graphene oxide has a higher specific surface area than graphene, but in this study, the specific surface obtained by modifying RF aerogel with 1% graphene was higher than previous studies using 1% amine-functionalized graphene oxide, and it also showed a higher adsorption capacity; in addition, graphene compared to graphene oxide and meta-phenylenediamine are more accessible and more cost-effective. In this study, graphene sheets were properly formed in the porous structure of the aerogel. The purpose of using graphene in aerogel modification was to increase the removal percentage, increase the adsorption capacity, increase the specific surface area and increase the use of adsorption mechanisms in order to further remove the minocycline antibiotic.

## Methodology

### Materials

Within the framework of this investigation, an aqueous solution of formaldehyde weighing 37% was harnessed, where methanol constitutes 11% of its weight, acting as a preventive factor, and manufactured by KBR (India). The procurement of resorcinol (98%) was accomplished through Sigma-Aldrich (Germany). The catalytic agent employed for the aerogel synthesis was sodium hydrogen carbonate (NaHCO_3_), procured from DUKSAN (South Korea). Minocycline, an integral component, was acquired from Hakim Pharmaceutical Co. (Iran). Acetone with a purity level of 99% from Dr. Mojallali’s Inc. (Iran), sulfuric acid with a concentration of 97–98% from Merck (Germany), oxygenated water with a purity of 30% from Merck (Germany), methanol from Merck (Germany), hydrochloric acid (HCl) at a concentration of 0.1 M from Merck (Germany), double distilled water, and natural graphite in flake form exhibiting an average dimension of 300 μm and a density of 2.3 g/cm^3^ with a purity of 98% from Superior Graphite Co. (China), were also used in this research.

### Synthesis of resorcinol formaldehyde (RF) neat aerogel

In the process of crafting pure RF aerogels, the initial step involved the dissolution of 6.25 g of solid resorcinol particles within 21.8 g of double-distilled water, employing the assistance of a magnetic stirrer. Following this, a quantity of 8.3 ml of 37% formaldehyde was introduced into the mixture. Sequentially, an addition of 50 ml of double-distilled water was combined with 1 g of sodium hydrogen carbonate. Catalyst reactions were carried out in a solution diluted with water to improve mixing and alleviate the high activation energy of the catalysts (resorcinol/catalyst = 400, resorcinol/water = 2). Hence, sodium hydrogen carbonate was then added to 0.015 g (0.755 mL) water, and the resulting solution was mixed for 15 min using a magnetic stirrer. The subsequent step involved transferring the solution into a polypropylene container. This mixture was then subjected to a temperature of 80 °C in an oven for 48 h. Following the natural drying process, any unreacted monomers remaining in the system after the solution had solidified were eliminated using a 4 wt% hydrochloric acid solution. Additionally, the solvent in the gel was substituted by distributing the solution over the gel using a syringe. Subsequently, the container was exposed to a temperature of 100 °C for 24 h. The ultimate gel product underwent multiple acetone washes to effectively eliminate any unreacted remnants. Lastly, the moist gel was placed in the oven at a temperature of 100 °C for 24 h under standard atmospheric pressure, resulting in the production of pure RF aerogel^40^.

### Synthesis of 1 wt% graphene-modified resorcinol formaldehyde aerogel (RF-G1)

**Figure 1:**
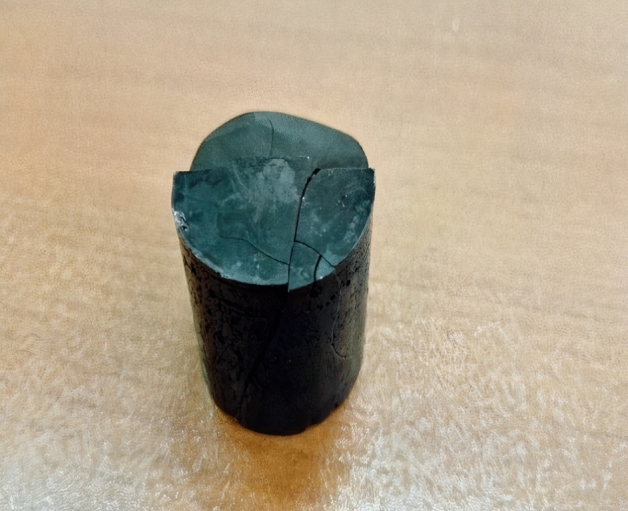
RF aerogel modified with 1 wt% graphene.

For the production of graphene-modified resorcinol formaldehyde aerogel (RF-G1) (resorcinol to catalyst ratio of 400, and resorcinol to water ratio of 2), a synthesis approach was employed. In this method, 0.38 g of graphene was introduced into 50 ml of double distilled water. The resulting suspension underwent initial agitation utilizing a magnetic stirrer, which lasted for 10 min. Next, to open the graphite layers and disperse the particles well, the suspension was exposed to ultrasonication for four 15-min periods. After ultrasonication, graphene was added to the resorcinol solution, which was prepared according to the aforementioned method. The following steps of preparing the RF-G1 were performed similarly to those of the neat aerogel. The synthesized modified aerogel is depicted in Fig. 1.

### Characterization

The examination of the samples that were prepared involved an assessment of their morphology. This evaluation was conducted through the application of field emission scanning electron microscopy (FESEM), employing the Tescan FESEM Mira 3 XMU instrument. The resulting micrographs were analyzed using Image J software. To delve into the characteristics of carbon atom hybridization and establish insights into the D-band and G-band, along with the ID/IG ratio, Raman spectroscopy was employed at a wavelength of 532 nm utilizing the Teksan equipment. For a precise determination of size, surface area, and porosity distribution, both the Barrett-Joyner-Halenda (BJH) and Brunauer–Emmett–Teller (BET) testing methods were conducted using the Belsorp mini II apparatus from Japan. Exploring the crystal structure of both unmodified resorcinol formaldehyde aerogels and aerogels modified with graphene, their crystallinity was scrutinized through X-ray diffractometry (XRD). This process was carried out using the Philips X'Pert instrument with parameters set at 40 mA current, 40 kV voltage, and a cobalt anode with a wavelength of 1.78901 Å. To comprehend the elemental composition, content distribution on the surface of the samples, and their elements, Thermo Finnigan's FlashEA1112 EDX-CHN test was harnessed. Besides, a helium pycnometer was utilized to determine skeletal density. To quantify the adsorption of antibiotics by the generated aerogels, a UV–vis instrument was employed from GBC in Australia, operating at a maximum wavelength of 357 nm. In order to analyze the regeneration capacity of prepared aerogel samples in absorbing antibiotics, samples were submerged in 5 wt% NaOH solution for about 5 h. After the desorption process, aerogels were removed from the solution, washed with distilled water, dried, and investigated again as adsorbents; then, their efficiency was compared to the initial samples.

### Adsorption experiments

The experiments for antibiotic adsorption were conducted in discrete batches, employing 100 mL Erlenmeyer flasks under controlled conditions of 25 °C. Stirring was facilitated through magnetic means at a rotation speed of 160 rpm. The flasks were covered with aluminum foil to minimize any potential degradation of the antibiotic due to exposure to light. In order to perform the adsorption experiments, aerogel samples were first grinded into the powder form with a laboratory mortar and suspended in the antibiotic solution. Accordingly, a higher interaction surface between the adsorbent and the antibiotic could be obtained. Within each sample container, an 8 mg portion of adsorbent material was present, along with a concentration of 40 mg/L of the antibiotic minocycline; this encompassed both the unmodified and modified aerogels. The pH of the solution was systematically adjusted using either 0.1 M NaOH or 0.1 M HCl, leading to a pH range spanning from 2 to 12, as measured by a pH meter. The interaction between the adsorbent compound and the antibiotic was sustained for 24 h. Subsequently, the liquid phase was separated from the solid material, and its content was quantified utilizing UV–Vis analysis. This approach was undertaken to ascertain the residual amount of antibiotic remaining in the solution after the interaction.

The assessment of the extent of removal (R%) and the adsorption capacity (qe, mg.g-1) was conducted using Eqs. (1) and (2), respectively:

1 $$\% R = \left[ {\frac{{C_{0} - C_{e} }}{{C_{0} }}} \right] \times 100,$$

2 $$q_{e} = \frac{{C_{0} - C_{e} }}{m}\, \times \,V,$$

In these equations, the initial concentration of the antibiotic (C_0_) in milligrams per liter (mg/L) was taken into account, along with the mass of the adsorbent (m) in milligrams (mg). Additionally, the equilibrium concentration (C_e_) of the antibiotic in milligrams per liter (mg/L) and the volume of the solution (V) in liters (L) were considered.

To facilitate the release of the adsorbent, both the RF and RF-G1 aerogels were employed, which absorbed the substances. These aerogels were subjected to a process of desorption by immersing them in a solution containing 5-weight percent NaOH for 5 h. Subsequent to this treatment, the recuperated samples underwent a sorting process and were subsequently rinsed with water. To prepare for the subsequent phase of the adsorption test, the dampened aerogels were subjected to drying; this was achieved by placing them in an oven set at a temperature of 100 °C, where they were allowed to remain for 24 h.

## Results and discussion

### Gelation mechanism of RF and RF-G1 aerogels

**Figure 2:**
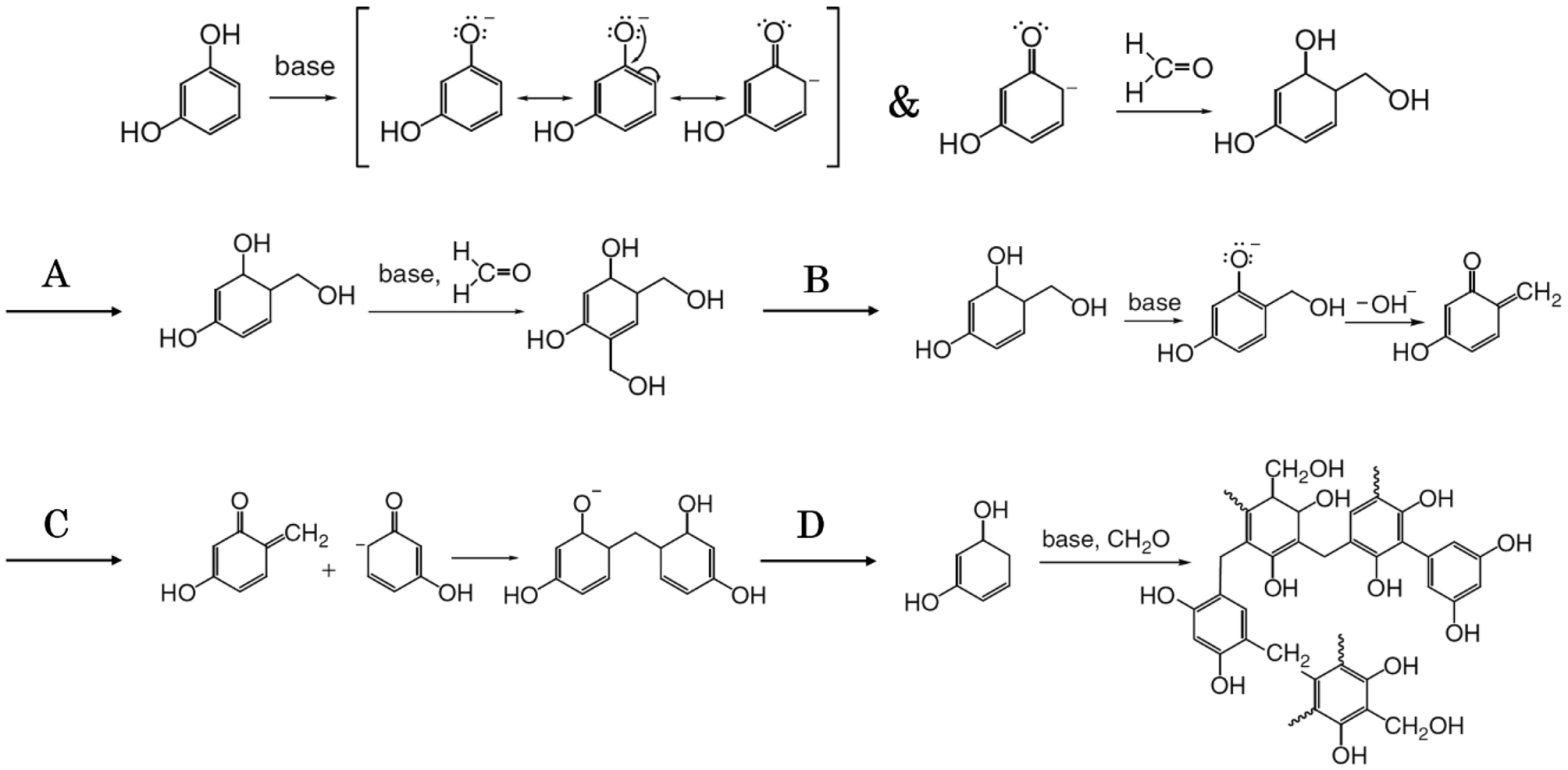
The function of sodium carbonate as a foundational catalyst in the crafting of RF aerogel and its underlying mechanism.

In order to better understand the effect of the structure of prepared aerogels on their performance, the mechanism of gelation in them was investigated. Within the samples that were meticulously prepared, the prominent interplay between resorcinol and formaldehyde was established through a gradual sequence of reactions. This process involved the conversion of molecules characterized by methylene-derived hydroxymethyl and methylene ether-bridged structures, eventually yielding derivatives that featured hydroxymethyl (CH_2_OH) groups. Resorcinol was deprotonated to the resorcinol anion in an alkaline media, as illustrated in Fig. 2. Due to the resonance, the electron concentration of resorcinol was increased at 4 (or 6) locations (Fig. 2a). The phenomenon of hydroxymethylation ensued as a consequence of an electron transfer proceeding from sites bearing negative charges to carbonyl carbon atoms within formaldehyde molecules, exhibiting a partial positive charge. As a result of this hydroxymethylation, other sites on the resorcinol structure became activated. Moreover, an excess supply of formaldehyde led to the occurrence of dihydroxymethylation, as depicted in Fig. 2b. The interaction between the O-quinone methide intermediate, the O-quinone methide intermediate, and the O-quinone methide intermediate becomes extremely unstable when the alkaline catalyst deprotonated hydroxymethylated resorcinol (Fig. 2c). The creation of O-quinone methide entities led to the establishment of robust connections with other molecules of resorcinol, thereby reinforcing the methylene linkage, as illustrated in Fig. 2d. This phenomenon resulted from the presence of o-quinone methides, coupled with elevated concentrations of electrons located at the 2-, 4-, and 6- positions of the resorcinol ring. This process rendered these positions more reactive compared to the phenol group. Given the availability of active sites on resorcinol molecules or clusters of resorcinol formaldehyde (RF), the potential for an extended condensation process increased substantially. Alkaline-catalyzed RF resins were developed as a result. The reaction produced many methylene-bridged novolac compounds. The heavily cross-linked clusters were the product of this multi-step procedure. Upon the culmination of these sequences, the colloidal particles merged, giving rise to a cohesive and interconnected framework. This structure showcased a circular arrangement reminiscent of pearl particles, effectively occupying the major portion of the aqueous medium. The production of RF-G1, both in its unaltered state and in conjunction with an aqueous mixture containing graphene, was executed utilizing sodium carbonate (as catalyst) during the process of sol–gel polymerization of resorcinol and formaldehyde. Subsequently, the resulting wet gels underwent a phase of natural drying at ambient room temperature, as depicted in Fig. 2. Graphene plates were created using the traditional sol–gel process in modified graphene materials. They were created as a strong adsorbent besides the porosity of the modified materials. As previously stated, the development of RF aerogel and RF-G1 gel was the result of two key reactions: (I) the formation of resorcinol hydroxymethyl derivatives and (II) the formation of methylene (–CH_2_–) bridges by the condensation of specific derivatives^40^.

**Figure 3:**
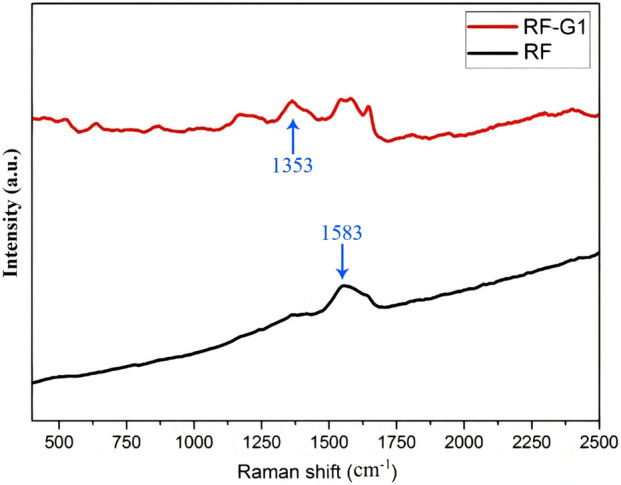
Raman spectra of neat and modified RF aerogels.

The formation of layers and structural flaws resulting from the production of neat and modified resorcinol formaldehyde aerogels was investigated using Raman spectra. The Raman spectra predominantly displayed two strong and broad peaks at 1353 cm^–1^ and 1583 cm^-1^, which were associated with the carbon D-band and G-band^56^ (Fig. 3); respectively. Nevertheless, the relative strength of these bonds was different. The system’s carbon sp^2^ hybridization or graphene structure gave rise to the G-band, whereas carbon sp^3^ hybridization, which resulted from flaws in the graphene crystal structure, gave rise to the D-band. This abnormality was measured using the I_D_/I_G_ (intensity ratio of the D-band to the G-band) ratio^57^. The escalation in the I_D_/I_G_ ratio corresponded to the emergence of hydroxyl functional groups within the system. This shift might be attributed to the augmented presence of sp^2^ regions^58^. Furthermore, the generation of hydroxyl functional groups and the transition of carbon in the system from its graphene state (characterized by sp^2^ hybridization) to an oxide state (marked by sp^3^ hybridization) could also contribute to this phenomenon. The I_D_/I_G_ ratio also could show the different degrees of graphitization of the samples. The high degree of graphitization was attributed to amorphous carbon blocks, which were excellent for ion transport and chemical performance. The I_D_/I_G_ ratio of neat resorcinol formaldehyde aerogel was 0.73. Also, this ratio increased to 0.97 in the RF-G1, indicating that the defects in the structure and graphitization of the modified sample had increased.

**Figure 4:**
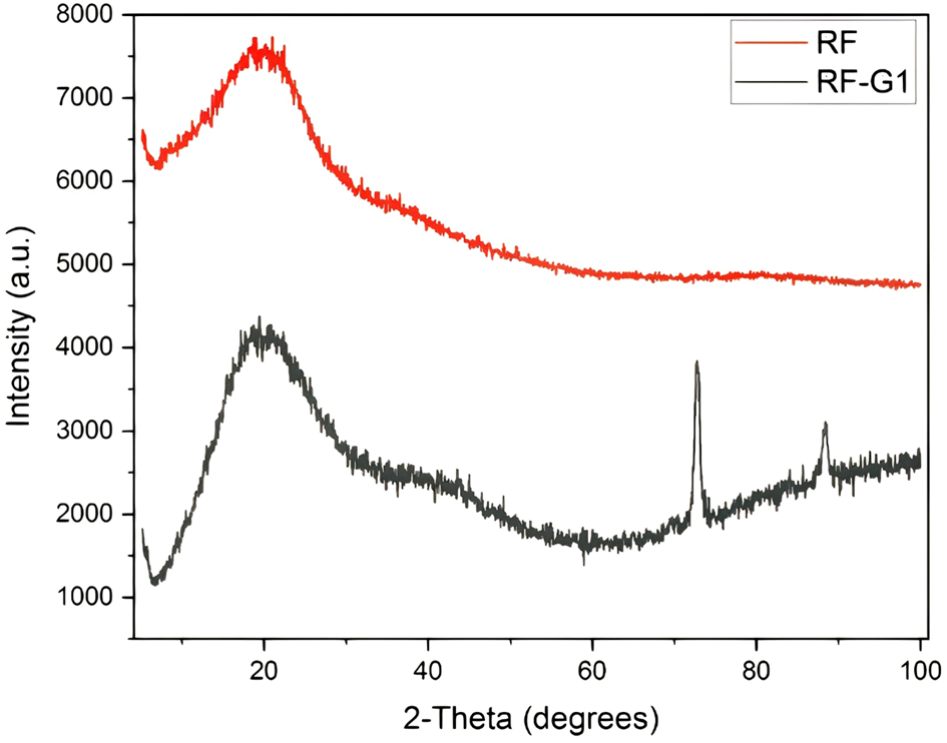
XRD patterns RF and RF-G1 aerogels.

The neat RF aerogel sample was entirely amorphous, without any crystal structure, as seen by XRD patterns in Fig. 3. According to JCPDS cards No. 162–75 and Fig. 4, 2θ = 44.37° and 83.17° illustrated the graphene particle graphite crystals for plates (112) and (101) in RF-G1. These peaks could demonstrate that the RF-G1 sample contained graphene. The distance between the plates was also measured using the Bragg equation, and the crystal size was determined using the Scherer equation. In these equations, θ indicated the reflection angle, λ showed the X-ray wavelength (1.5418 Angstrom), β showed the half-height peak thickness (FWHM), K demonstrated the shape coefficient, and L was the crystal size. The dimensions of the crystals along their longitudinal and radial orientations were represented by L_c_ and L_a_, with corresponding values of 0.9 and 1.84 for the parameter K. The presence of larger-sized crystals could lead to an increase in surface area, facilitating smoother crystal penetration and more effective absorption of antibiotics. This phenomenon was subsequently substantiated by the outcomes of the UV–Vis testing.

**Figure 5:**
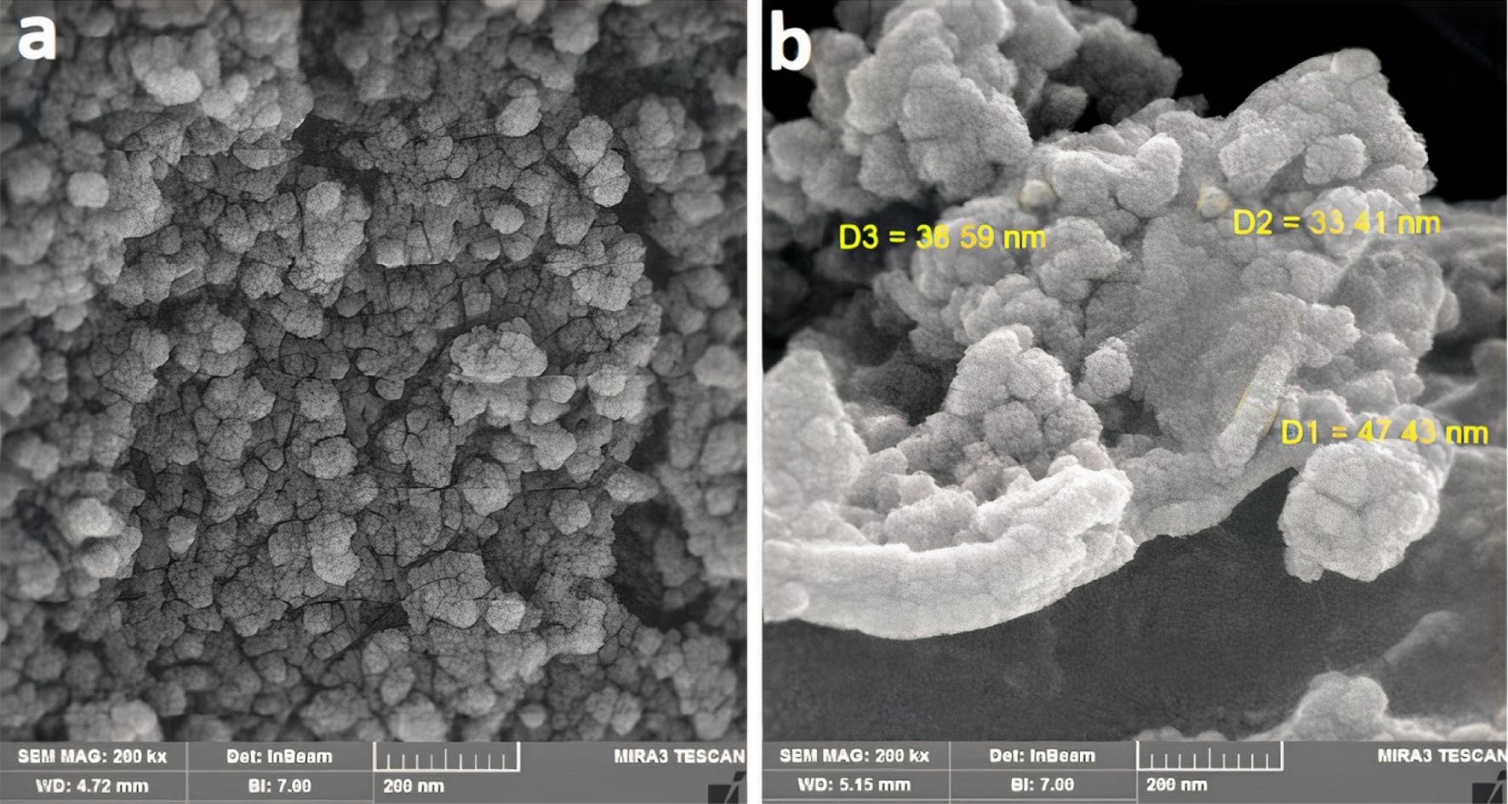
FESEM images of samples; (**a**) RF, (**b**) RF-G1 at 200k $\times$ magnification.

FESEM micrographs of neat and modified RF aerogel samples are shown in Fig. 5 at 200 k $\times$ magnification. The micrograph of RF aerogel containing graphene demonstrated the presence of a multi-nanoparticle, interconnected network, and a three-dimensional porous structure. The measurements of particle dimensions achieved in the experiments, along with the establishment of nanoporous characteristics, were conducted at the nanoscale level. The resultant structural appearance was found to have a connection with the aerogel samples’ formation methodology. This outcome could potentially be influenced by factors such as the ratios of resorcinol to water, resorcinol to catalyst, and resorcinol to formaldehyde^59^. In the resulting structure, small spherical particles were linked together in a pearl-like network. The structure of RF aerogel was formed into a neat cluster and was made up of tiny linked spheres. On the aerogel matrix, interconnected microspheres were arranged fairly equally.

**Figure 6:**
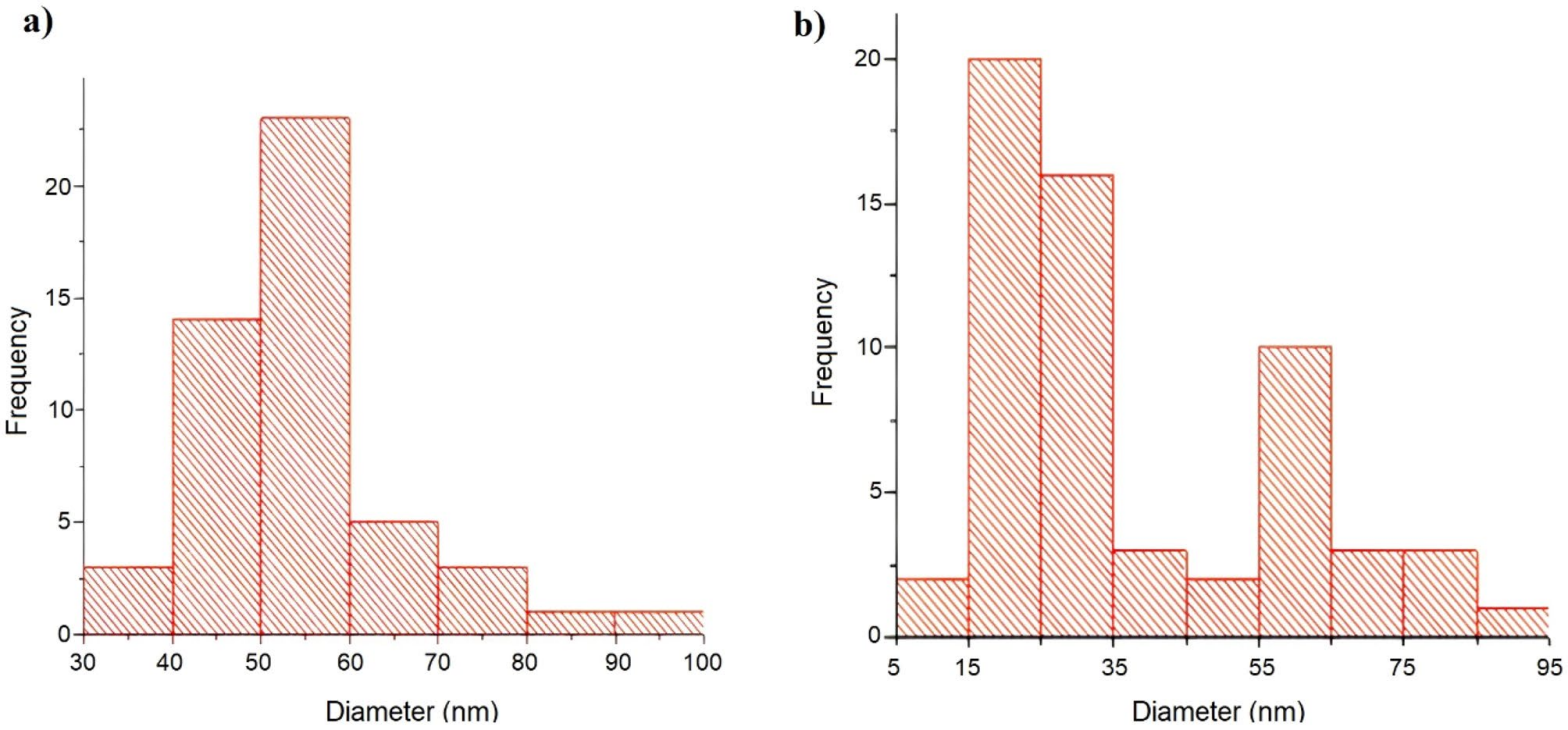
Particle size distribution of (**a**) RF and (**b**) RF-G1.

Particle size was calculated using the Image J program employing the Gaussian model. Figure 6 demonstrates the distribution of particle sizes histogram of samples generated. Each particle was measured in four distinct orientations. The resorcinol/catalyst ratio could be one of the factors affecting the aerogel particle size. As illustrated in Fig. 6a, the selection of NaHCO_3_ as the catalyst along with a resorcinol-to-catalyst ratio of 400 resulted in particle dimensions falling within the range of 30 to 100 nm. The mean particle size was quantified at 52.4 nm, and the corresponding standard deviation was calculated to be 7.4.

The configuration of RF-G1, as depicted in Fig. 5b, deviated from a clustered arrangement. The analysis revealed that the average particle size was 25.5 nm, with a size range spanning from 5 to 95 nm. This outcome was characterized by a standard deviation of 6.9 nm. These observations pointed towards a reduction in the size of spherical particles, contributing to a denser and more compact structure formation. This transformation was substantiated by the histogram showcasing particle size distribution, which clearly indicated that the particle dimensions were smaller compared to those of the unmodified aerogel. According to the histogram data, the adjusted sample comprised smaller particles. This adjustment consequently led to a higher specific surface area in comparison to the neat RF aerogel. Moreover, there was an increased particle count per gram of material in this case. Subsequent verification via the BET test further supported these findings. The resulting meso- and macro-pores were appropriate for eliminating minocycline antibiotics, which have a particle size range of 20–70 nm.

Except for hydrogen, every element present on the surface of the synthesized aerogels was quantified using the EDS test. The presence of C, H, and N elements on the surfaces of the synthetic aerogels was determined by the CHN/O test. These two analyses were combined to determine the weight percentages of all the components on the surfaces of modified and neat aerogels.

One of the mechanisms of the elimination of antibiotics from the media using aerogels is the formation of hydrogen bonds across the aerogel matrix with antibiotics^55^. F, O, and N elements form hydrogen and could bond with each other. According to the findings, the RF-G1 sample was more likely to create a hydrogen bond with minocycline antibiotics when compared to the other investigated sample based on the content of the element. Furthermore, given the nitrogen and oxygen constituents present on the surface of minocycline, which have the potential to engage in hydrogen bonding, the increased hydrogen content within the system could significantly augment the probability of hydrogen bond formation between antibiotics and the synthesized aerogels. The outcomes of the EDS and CHN/O tests are detailed in Table 1. Accordingly, the integration of graphene into the RF aerogel structure emerges as a judicious selection, effectively enhancing the propensity for hydrogen bonding and consequently promoting the enhanced adsorption of minocycline antibiotics.

**Table 1:** Elemental measurement of samples made using EDS and CHN/O tests.

	C (%)	H (%)	N (%)	O (%)
RF	72	4	–	24
RF-G1	58	10	–	32

Table 2 provides a concise summary encompassing the density and porosity characteristics of both RF and RF-G1 aerogels. The physical measurements and weight of the samples were used to compute the bulk density, and a helium pycnometer was used to calculate skeletal density. According to the formula below, the bulk density (ρ_b_) and skeletal density (ρ_s_) were used to calculate the porosity (P) of the synthetic aerogels:

**Table 2:** Porosity and density of RF-G1 and RF aerogel samples.

Sample	${\rho }_{b}$(g/cm^3^)	${\rho }_{s}$(g/cm^3^)	P (%)
RF	0.02 ± 0.29	0.03 ± 1.94	85
RF-G1	0.01 ± 0.35	0.03 ± 3.40	89

3 $${\text{P}}=\left[\frac{\frac{1}{{\rho }_{b}}-\frac{1}{{\rho }_{s}}}{\frac{1}{{\rho }_{b}}}\right]\times 100.$$

The density of neat RF aerogel was lower compared to the RF-G1 specimen and by adding graphene to the RF aerogel, the density of the aerogel was altered; in other words, using graphene in the modified sample increased the density.

Moreover, the BET technique was employed to gauge the influence of the sample preparation variables on both the specific surface area and the size of the pores; this involved a thorough alteration of the underlying structure. The analysis of the diameter distribution, pore size, and volume was carried out using the BJH technique. The volumes of pores were determined at the micro and mesoscales utilizing the T-PLOT technique. The data obtained from these tests are displayed in Table 3. Within the context of BET analysis, it becomes feasible to acquire the complete pore composition, revealing the aggregate content of both mesopores and micropores. Determination of the micropore volume was accomplished through the utilization of the T-plot diagram, hence the measurement furnished within this segment pertains exclusively to the micropore volume encompassing the sub-2 nm range.

**Table 3:** Parameters corresponding to the specific surface area, pore volume, and pore dimensions concerning both RF and RF-G1 aerogels.

Sample	Specific surface area $(m^{2} /g)$	Pore size (nm)	$V_{mic} \,$ $(cm^{3} /g)$	$V_{mes}$ $(cm^{3} /g)$	$V_{total}$ $(cm^{3} /g)$	$S_{mes}$ $(m^{2} /g)$	$S_{ext}$ $(m^{2} /g)$
RF	96	47	0.015	0.95	0.965	15	81
RF-G1	260	55	0.08	0.28	0.36	100	160

**Figure 7:**
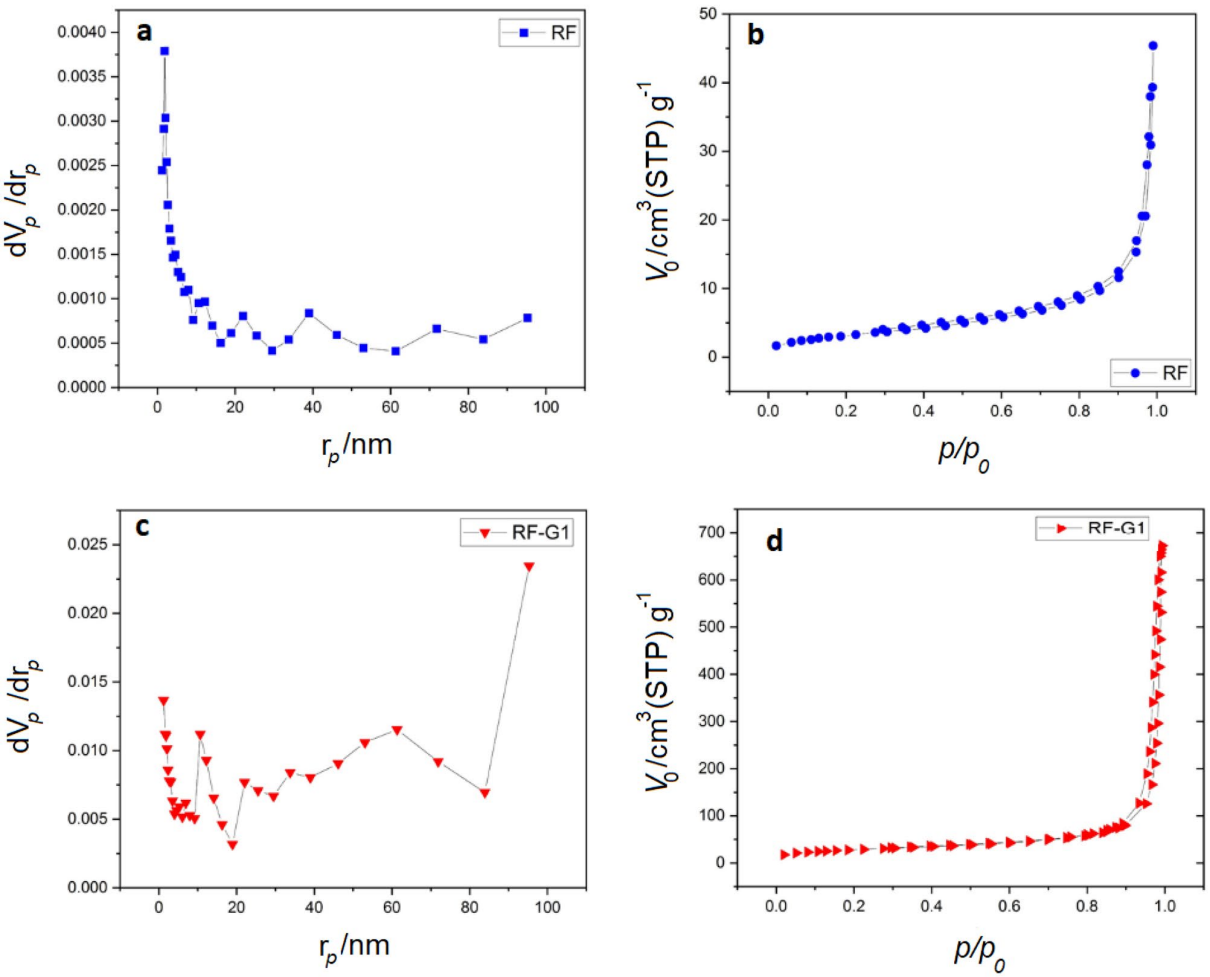
(**a**,**c**) The entire volume of pores in various porosity sizes: (**b**,**d**) the adsorption–desorption diagram of RF and RF-G1 aerogels.

Table 3 illustrates the augmentation of the specific surface area through the incorporation of graphene into the pristine RF aerogel. Figure 7b and d present the distributions of pore diameters in the unmodified and graphene-modified RF aerogels, alongside the N_2_ adsorption–desorption isotherms. The adsorption isotherms of the resultant aerogels adhered to the classification of type IV, as per the IUPAC categorization. The overall distribution of pore sizes in Fig. 7 demonstrates that meso, micro, and macropores are included in the produced aerogels and vary in size from 1.21 to 100 nm. Hysteresis patterns were evident in all the N_2_ adsorption isotherm graphs. This phenomenon, frequently associated with the capillary density within mesoporous frameworks, manifested within the multilayer domain of physical adsorption isotherms. The presence of hysteresis in the adsorption–desorption plots indicated the presence of mesopores within the generated materials. The adsorption–desorption diagrams (Fig. 7b,d) indicate that micro-scale pores were the predominant type of pores in prepared samples. Figure 7 also shows that the loop formed in the adsorption–desorption diagrams of RF-G1 was related to the H3 classification, which showed that the mesopores were mainly rooted in the layered pores between the graphite layers.

In resorcinol-based aerogels, resorcinol/catalyst and resorcinol/water parameters are two effective parameters in the aerogel structure. In this study, according to previous experiments and optimization, a resorcinol/catalyst ratio of 400 was used, and the resorcinol/water ratio was kept at 2, which was shown to have the highest specific surface area.

### Minocycline antibiotic removal mechanisms using graphene-modified aerogel

The antibiotic minocycline interacts with RF-G1 in a π-π stacking manner. The π system is a part of this non-covalent interaction type. The π system can be in connection with one molecule or another π system and has many electrons. π-stacking is another name for aromatic-aromatic interactions^60^. This effect is brought about by stacking a pair of aromatic groups, as seen in graphene and cyclic architectures of the minocycline antibiotic. Enhancing the count of rings within the antibiotic molecule expedites the adsorption process onto carbonaceous substances due to the acceleration of π rings within its hexagonal molecular structure^61^. Conjugated benzene rings are also a feature of minocycline. The primary mechanism of adsorption between minocycline antibiotics and graphene involves electrostatic interactions, predominantly attributed to π-π stacking within an electron donor–acceptor framework^62^. Furthermore, graphene harbors various oxygen atoms and conjugated π electrons within its structure, manifested through hydroxyl, epoxy, and carboxyl groups^63^. Hence, the interaction facilitated by the modified aerogel surface and the ring structure of minocycline could readily involve π-π stacking.

Another reason for the adsorption of minocycline antibiotic is the formation of complexes between different types of antibiotics, including cationic, anionic and zwitterionic bonds, and the surface of the modified aerogel sample, which were dominant in acidic conditions of cationic antibiotic species and with an increase, pH value and the ratio of cationic species decreased and zwitterionic species increased. Above pH 7.5, the content of zwitterionic species began to decrease and anionic species increased. In general, increasing the pH increased the negative charge on the surface of minocycline and RF-G1, which led to electrostatic repulsion and a decrease in the amount of adsorption in alkaline conditions. In acidic conditions, a positive charge of the antibiotic formed in the solution, and it created a negative charge on the surface of the neat and modified aerogel, and these two species attract each other with cationic and anionic charges. When the antibiotic species were zwitterions, the adsorption capacity of the modified sample does not alter despite variations in minocycline charges^64^.

Some strong adsorption linkage procedures across graphene and minocycline are as follows: π-cation bond, Van der Waals forces (London scattering and perpetual induced dipole–dipole forces), and π-π electron-donor–acceptor across the determined graphene π electrons and amine proton groups^65^. One could assert that the effectiveness of adsorbing minocycline antibiotics onto RF-G1 experienced a substantial influence from electrostatic interactions. The van der Waals forces’ potency is defined by the specific van der Waals index between an adsorbent surface and its contact surface. A graphene surface is an extraordinarily high van der Waals index carbon adsorbent^66^. Due to the plate-shaped ring structure of minocycline molecules, there are likely significant van der Waals interactions across the graphene surface and minocycline molecules acting as the adsorbent. Non-covalent mechanical adsorption comes into play through the occurrence of π-π stacking and van der Waals forces. The enticed minocycline molecules need to be positioned proximal to the graphene surface, resulting in head-to-head configurations. This arrangement amplifies both van der Waals forces and the interactions between donor–acceptor π electrons^67,68^. The interconnected amino group maintains a connection with the graphene surface via a π-cation bond, ensuring effective interaction while upholding a non-interfering tail-to-tail configuration. In contrast, the alignment of minocycline rings involves their coordination with the protons of the enol, phenol, and amide groups. For the creation of a π-H complex, the proton must stand perpendicular to the ring surface. This particular orientation renders the formation of π-H bonds with the graphene surface unattainable. Hence, an efficient way for nitrogen atoms to bond to pristine RF and modified aerogels was via sharing electron pairs and creating complexes, which facilitated the adsorption of the antibiotic on the aerogel surfaces.

Accordingly, according to the size of the pores created in RF-G1 and the average size of minocycline antibiotic particles (approximately 25.5 nm) for adsorption by the pore filling method, the size of the pores created in the modified sample had to be on a macro and meso scale, which according to the data obtained from the FESEM test, histogram of particle size distribution, BET and BJH, which emphasized the presence of major meso-pores, confirmed the possibility and dimensional favorability of absorption of minocycline antibiotic by RF-G1 by pore-filling mechanism.

**Figure 8:**
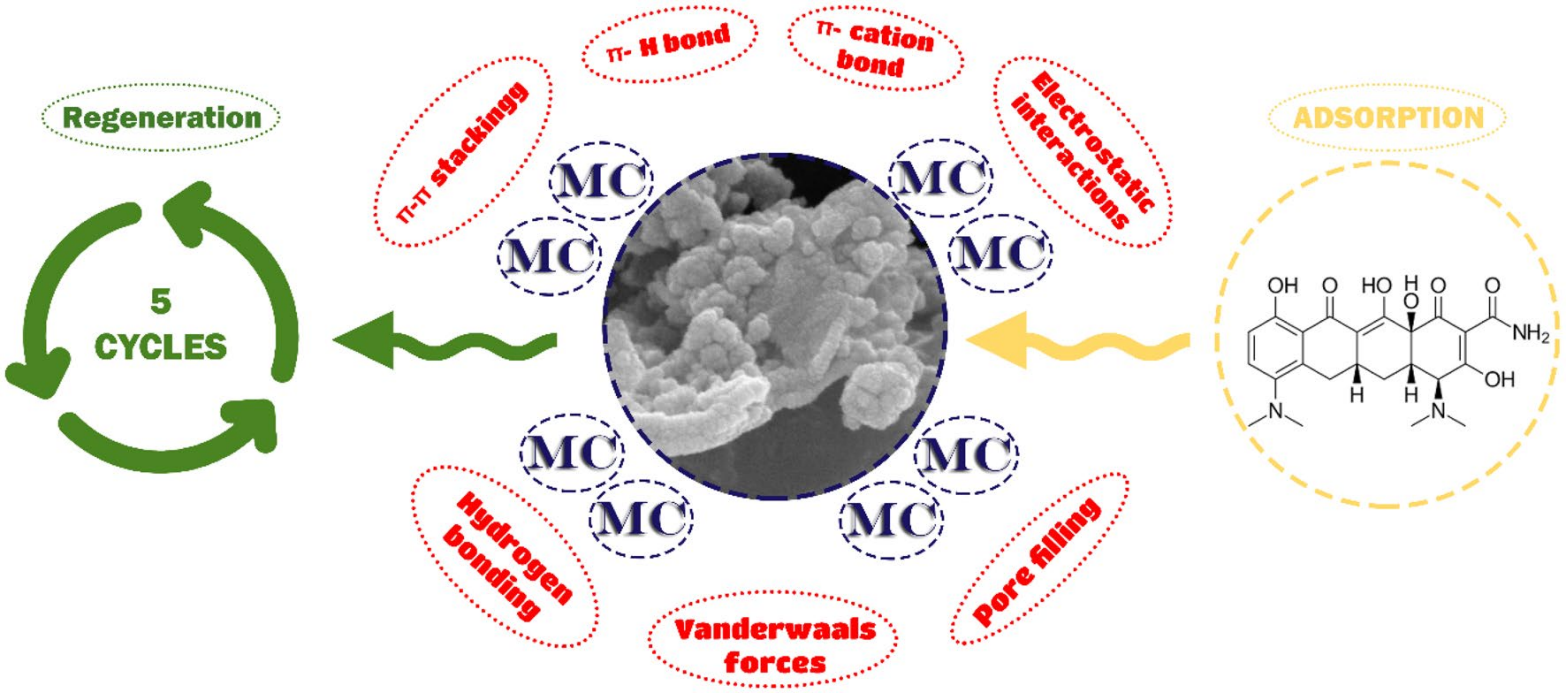
The adsorption mechanisms of minocycline antibiotic by the RF-G1.

The minocycline antibiotics can be adsorbed by the RF-G1 through different mechanisms including through π–π stacking, π-cation bond, π-H bond, hydrogen bonding, electrostatic interaction, and pore-filling mechanism as shown in Fig. 8.

### Antibiotic removal performance

#### The effect of pH

In general, one of the key markers of water quality that might impact the efficiency of different separation processes, such as adsorption, is the pH of the solution^69^. Hence, assessing the removal efficiency of modified aerogels from minocycline at various pH settings was required. As the pH of the media varies, so does the distribution of minocycline species. Hence, the UV–vis test was used to determine the ideal pH that may lead to the most efficient adsorption. The pH level where the maximum adsorption rate takes place is known as the optimal pH. The pH level influences the loading of the minocycline antibiotic, which can assume zwitterionic, cationic, or anionic forms under neutral, acidic, and alkaline conditions correspondingly^70^. At pH above 7.85, the antibiotic minocycline also manifests as an anionic species^71^. A reduction in adsorption could result from the anionic dissociation of the antibiotic minocycline and electrostatic repulsion between the negative charges of the surface of the neat or modified samples when the pH of the antibiotic species' solution was high. Moreover, the RF-G1 sample structure contained conjugated structures, unsaturated double bonds, and hydroxyl groups that were ideal for the adsorption of antibiotics like minocycline.

**Figure 9:**
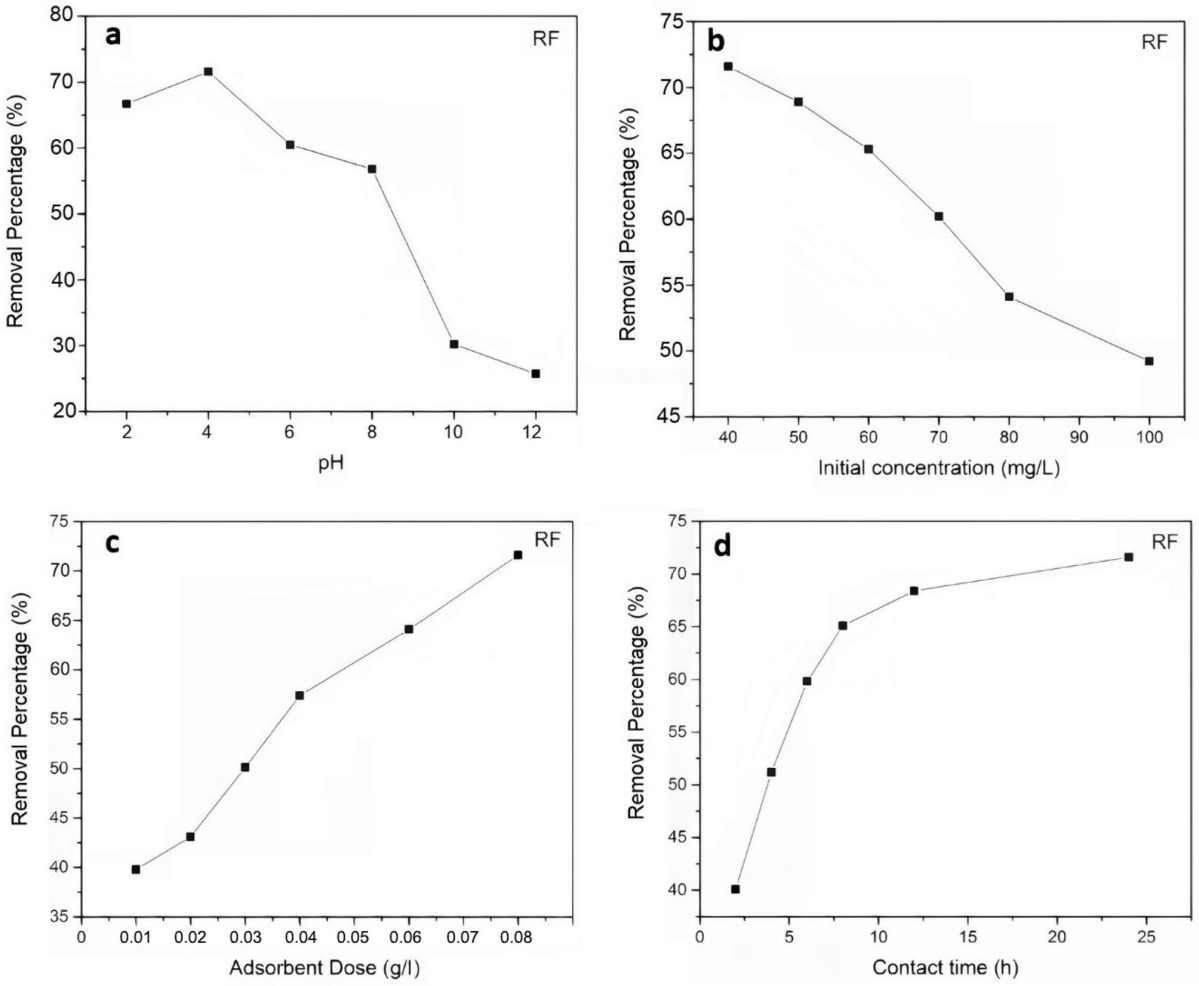
In-depth analysis of the influence of pH, adsorbent quantity, initial concentration, and contact time adsorbent and adsorbate on the unmodified RF aerogel.

**Figure 10:**
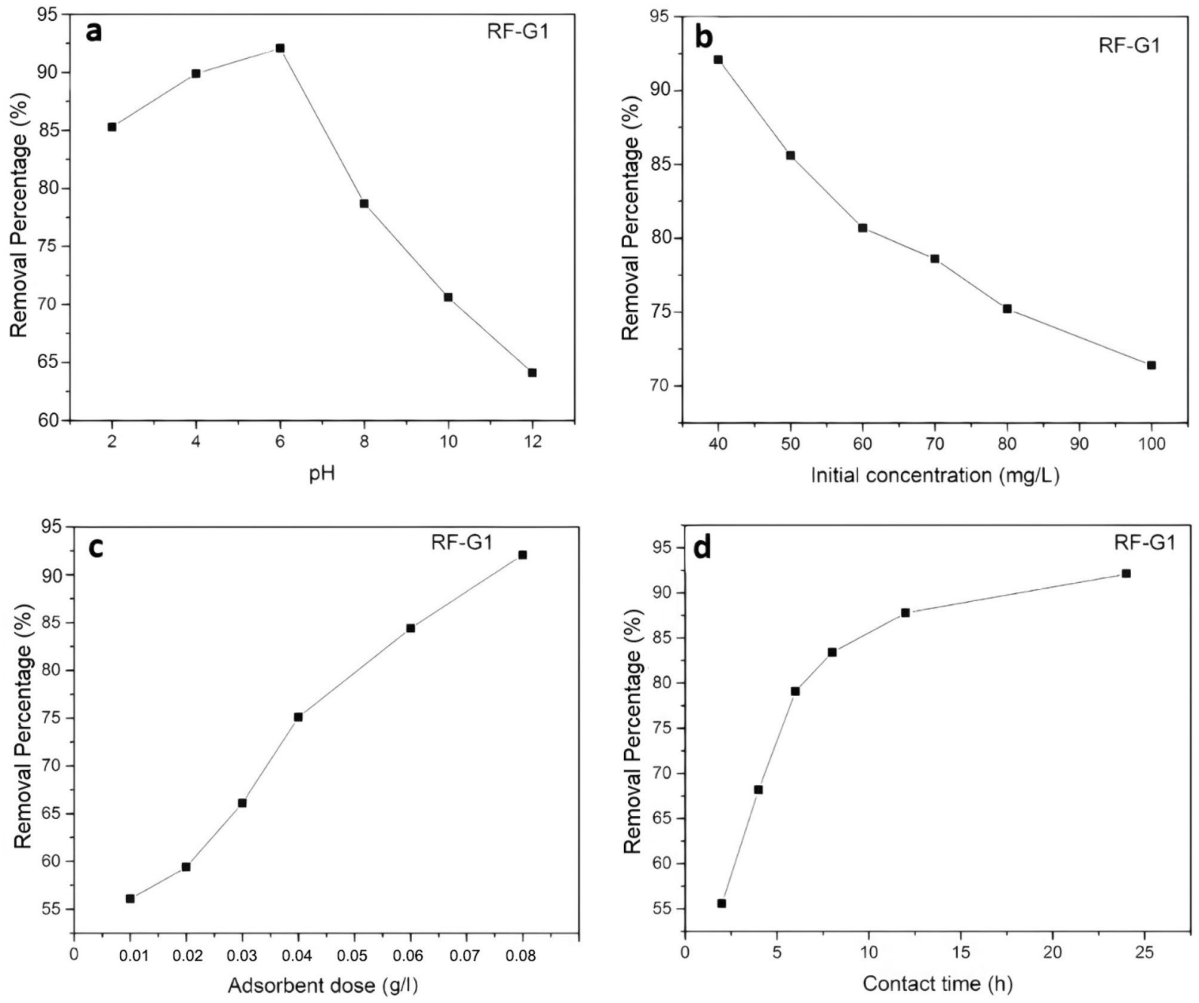
Effect of pH, adsorbent dose, initial concentration, and contact time adsorbent and adsorbate for RF-G1 aerogel.

The pH of the cationic species of the antibiotic minocycline decreased as the zwitterionic and anionic species increased, and the electrostatic repulsion in the alkaline medium caused its reduction. The rate of antibiotic elimination by plain and modified RF aerogel in an acidic medium was generally enhanced in higher pH levels, as shown in Figs. 9a and 10a.

#### The initial concentration of antibiotic

To achieve optimal adsorption conditions, an exploration into the impact of the antibiotic’s solution concentration on removal efficiency was conducted. The outcomes of this investigation are visually depicted in Figs. 9b and 10b. As the initial concentration of antibiotic increased, the rate of adsorption decreased. In higher concentrations of minocycline antibiotics, more antibiotic molecules had to compete for adsorption sites in aerogels, by which the driving force for the removal of minocycline antibiotics was generated. Besides, with increasing initial antibiotic concentration, active sites were saturated; since the adsorption was irreversible, its rate and consequently capacity decreased in higher initial concentrations.

#### Adsorbent dose

Another factor that can affect the behavior of adsorbent materials for the removal of antibiotics or other materials is their dosage^38^. In general, for processes that are based on the active sites of particles, the adsorption efficiency can increase with increasing the adsorbent dose, and then decrease due to a reduction in the active sites and decreased surface area, caused by the agglomeration of particles^72^. However, for aerogels, an increased adsorbent dose can lead to higher adsorption^73^. In this study, increasing the dose of neat and modified RF aerogel led to increased removal of minocycline antibiotics due to the increase in accessible surfaces for adsorption or adsorbent sites in the three-dimensional adsorbent network (Figs. 9c and 10c).

#### Contact time between adsorbate and adsorbent

In this study, the removal rate improved with increasing the contact time of adsorbate and adsorbent (Figs. 9d and 10d). At first, numerous empty adsorption locations on the absorbent existed which increased the rate of adsorption; in other words, the capacity to participate in removing minocycline antibiotics was higher, making the removal of antibiotics faster. Removal also occurred quickly in the early stages after 8 h of absorbent and adsorbate contact. After 8 h, the rate decreased, and the minocycline removal rate was nearly constant, as the adsorbate molecules had to compete for empty sites due to the irreversibility of the adsorption and the saturation of active sites.

### Regeneration

**Figure 11:**
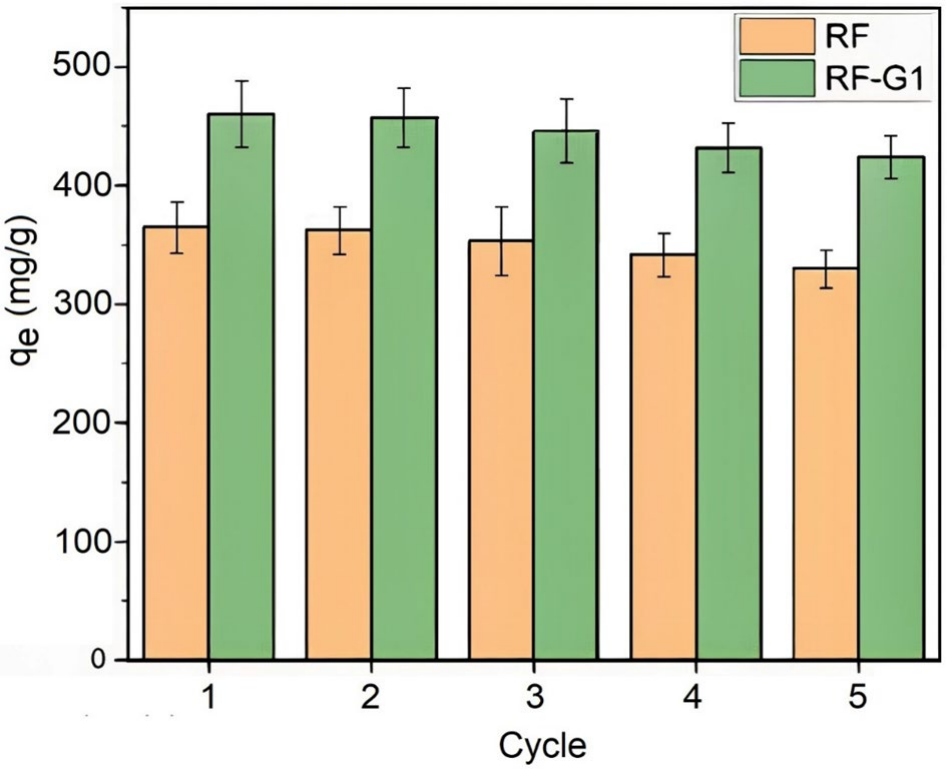
Regeneration capacity for RF and RF-G1. Error bars represent an SD (n = 3).

Adsorbents should have the ability to be recovered after performing the adsorption procedure to increase their cost efficiency. In the present investigation, the adsorption of RF and RF-G1 aerogels were studied for five consecutive cycles; the results are shown in Fig. 11. The adsorbents displayed periodic behavior; for RF-G1 samples, throughout five cycles, the equilibrium adsorption capacity decreased about 5%, from 2 to 8%. It indicated that the occupied adsorption locations were irreversibly occupied. However, when juxtaposed with the unmodified aerogel, the decline in adsorption capacity exhibited a more restrained trend for the aerogel infused with graphene.

According to other studies, Saghir et.al observed that ZIF-8 was adsorbed minocycline for up to 4 cycles and > 90% of the initial adsorption capacity (up 500 mg/g) was retained^74^. Also, in another study, Saghir et.al observed that for Ag@ZIF-67, 75% minocycline removal was achievable after 5 cycles^71^. Ying Zhang et.al observed Fe3O4@MIL-68(Al) maintained a good adsorption capacity and recovery efficiency remained > 95% after 3 cycles^37^. This contrast could underscore the cost-efficiency, efficacy, and prospective utility of RF and RF-G1 aerogels in the removal of minocycline antibiotics from aqueous solutions.

### Kinetics and isotherm of adsorption

**Figure 12:**
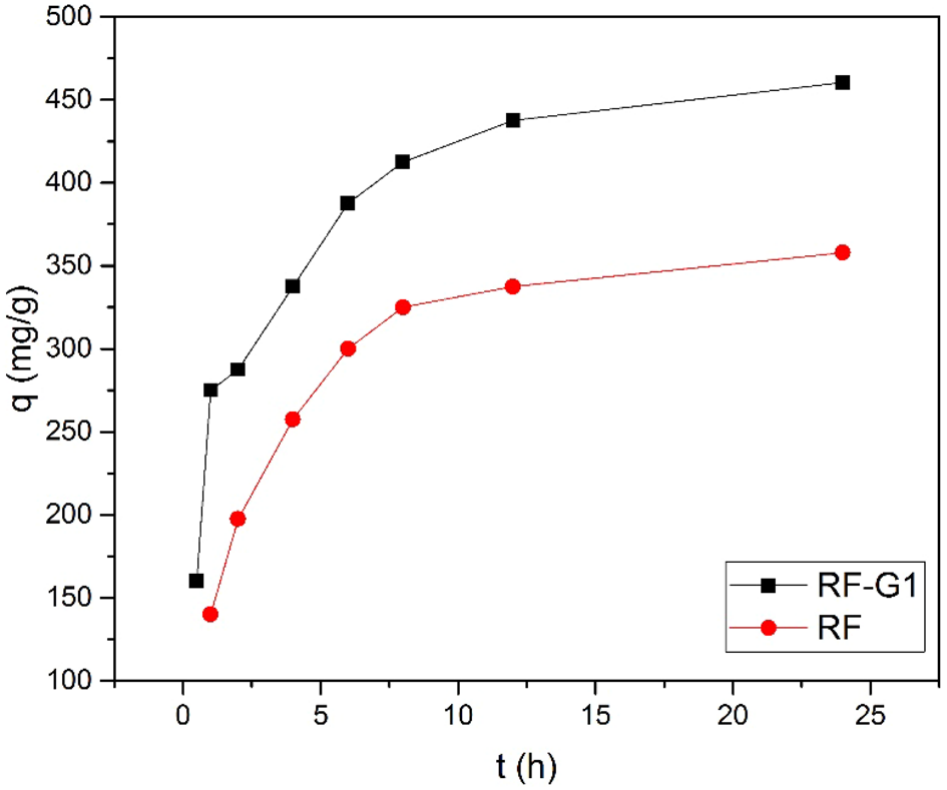
Nonlinear pseudo-second-order adsorption kinetics.

Table 4 and Fig. 12 present the computed kinetic parameters associated with the pseudo-second-order kinetics model for the adsorption of minocycline onto RF-G1 and RF. The adsorption kinetics of minocycline onto RF-G1 were scrutinized through the application of the following pseudo-second-order kinetics model:

4 $$\frac{{\text{t}}}{{{\text{q}}}_{{\text{t}}}}=\frac{1}{{{\text{k}}}_{2}{{\text{q}}}_{{\text{e}}}^{2}}+\frac{{\text{t}}}{{{\text{q}}}_{{\text{e}}}} ;{\text{h}}={{\text{k}}}_{2}{{\text{q}}}_{{\text{e}}}^{2},$$

where, q_t_ (mg.g^–1^) and q_e_ (mg.g^–1^) are the number of antibiotics adsorbed on the adsorbent surface at any time and the number of antibiotics adsorbed in equilibrium, respectively; k_2_ (g.(mg.h)^–1^) is the adsorption rate constant in the pseudo-second-order model; h is the initial rate of adsorption when t → 0. The model fitted well with the adsorption data for neat and modified aerogels, and the R^2^ values for RF-G1 were 0.99 and 0.99, respectively. These findings suggested that the fast adsorption of minocycline onto modified aerogel was a result of chemical adsorption. The adsorption rate constants were 0.002 and 0.005 g.(mg.h)^–1^ for RF and RF-G1 for minocycline removal, respectively. In addition, the values of adsorption capacity at equilibrium (q_e_) calculated by the model were 450.5 and 500 mg.g^-1^ for RF and RF-G1 for minocycline removal, respectively, indicating excellent compatibility with the values obtained from the experiments.

**Table 4:** Kinetic properties of minocycline removal utilizing RF and RF-G1 aerogels obtained by using a pseudo-second-order model.

Sample	Antibiotic	K_2_ (min^–1^)	h (mg.(g.min)^–1^)	R^2^	SSE
RF	Minocycline	0.002	405.9	0.99	22.5
RF-G1	Minocycline	0.005	1250	0.99	16

**Figure 13:**
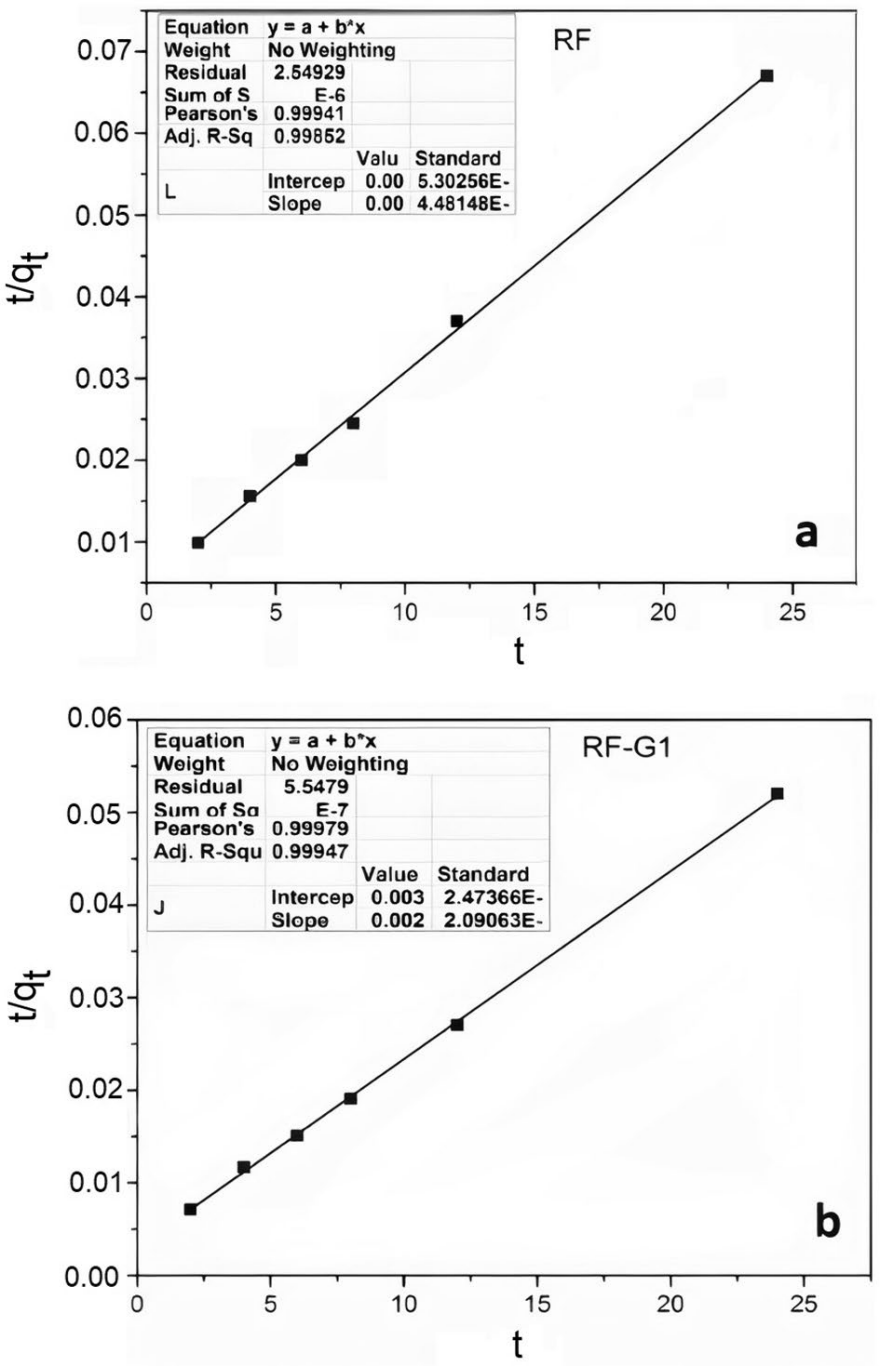
The outcomes obtained from fitting the empirical data to the pseudo-second-order model for minocycline adsorption: (**a**) RF and (**b**) RF-G1.

One of the reasons adsorption kinetic studies are performed is to measure the time required for the system to reach the equilibrium state. t0.5 and t0.95 are the time required to reach 50 and 95% saturated adsorption, respectively. According to Fig. 12, t0.5 and t0.95 were 1.6 h and 14.5 h for RF and for RF-G1 50 min and 13 h, respectively. Hence, according to the obtained results for the equilibrium studies, a time of 14 h was chosen to obtain the equilibrium adsorption isotherms^75–77^. In addition, the obtained model is shown in Fig. 13.

**Figure 14:**
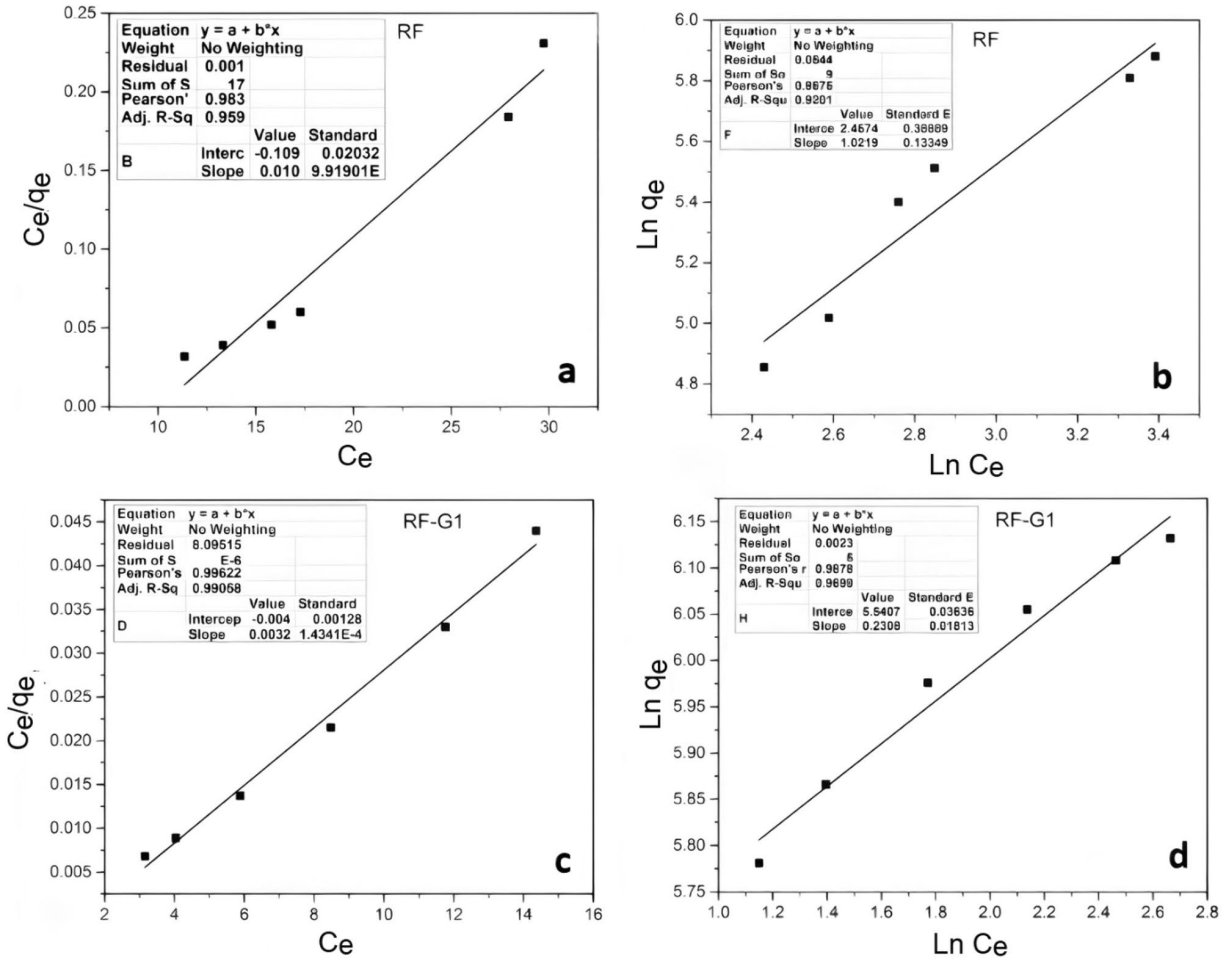
Experimental data fitting results for different isotherm models: (**a**,**c**) Langmuir model for RF and RF-G1, (**b**,**d**) Freundlich model for RF and RF-G1.

Adsorption isotherms encompass a series of mathematical formulations that elucidate the correlation between the equilibrium adsorption value in the solid phase (q_e_) and the equilibrium concentration of adsorbate in the liquid phase (C_e_), under constant temperature conditions. These isotherms play a pivotal role in the formulation of adsorption methodologies^78^. To fit the experimental data and for explaining the adsorption equilibrium, Langmuir and Freundlich's models, which are traditional adsorption models, were used (Fig. 14). The formulas for the Langmuir and Freundlich models are:

4 $$\frac{{{\text{C}}}_{{\text{e}}}}{{{\text{q}}}_{{\text{e}}}}=\frac{{{\text{C}}}_{{\text{e}}}}{{{\text{q}}}_{{\text{m}}}}+\frac{1}{{{\text{k}}}_{{\text{L}}}{{\text{q}}}_{{\text{m}}}},$$

5 $${{\text{Lnq}}}_{{\text{e}}}={{\text{Lnk}}}_{{\text{F}}}+\frac{1}{{\text{n}}}{{\text{LnC}}}_{{\text{e}}}.$$

In the context of this study, wherein q_m_ (mg.g^–1^), *n*, k_L_, and k_F_ denote the maximal theoretical adsorption capacity per unit weight of adsorbent, the Freundlich linearity index, as well as the Langmuir and Freundlich model’s adsorption constants correspondingly. For the adsorption process involving minocycline and RF, the Langmuir model exhibited a strong correspondence (R^2^ = 0.95), while a closely fitting relationship was observed for the RF-G1 system (R^2^ = 0.99). Moreover, the data yielded an acceptable agreement with the Freundlich model (R^2^ = 0.92) in the case of RF, and a similar trend was noted with RF-G1 (R^2^ = 0.96). Thus, the Langmuir model generally fitted well at certain concentration levels rather than high levels, indicating the limitations of the monolayer adsorption hypothesis.

In addition, the Freundlich model showed closer fitting at high concentrations, indicating chemical adsorption. Simultaneously, the Langmuir model calculated the maximum ideal adsorption capacity (q_m_) was obtained equal to 100, 312.5 mg.g^–1^.

Table 5 is a summary of the adsorption capacities and adsorption conditions of the minocycline antibiotic with using different adsorbents. This table also shows that the RF-G1 in the present study effectively and optimally removed the minocycline antibiotic from the aqueous solution. Results were also compared with other adsorbents in this field.

**Table 5:** Comparison of maximum adsorption capacities adsorbents of minocycline antibiotic.

Adsorbent [reference]	Adsorption capacity (mg.g^–1^)	Adsorption conditions
Fe_3_O_4_/OMC	193.5	293 K, 0.16 h, pH = 8
ZIF-8^74^	530.9	323 K, 24 h, pH = 6
Fe_3_O_4_@MIL-68(Al)^37^	248.1	298 K, 2.66 h, pH = 6
Ag-ZIF67^71^	994.7	313 K, 3.33 h, pH = 8
LDHSMgAl-LDH/C-AlO(OH)/C	302	493k, 70 h, pH = 7
CeO_2_^79^	6.9	298 K, 4 h, pH = 4.4
MgFe_2_O_4_/γ-Fe_2_O_3_^80^	119	298 K, 48 h, pH = N/A
Wated FeNiR Sludge^81^	1.9	303 K, 2h, pH = 4
RF-G1 aerogel (this study)	460.5	298 k, 24 h, pH = 6

## Conclusions

In this study, a resorcinol formaldehyde (RF) aerogel was synthesized and modified in-situ using 1 wt.% graphene (RF-G1) during the synthesis step. Characterizations showed that the RF aerogel and modified sample had a porous structure with meso- and macro-pores, which were formed in the modified aerogel by graphene sheets. Aerogel modification with graphene increased the removal capacity of minocycline antibiotic up to 92.1% by electrostatic interaction, pore filling, van der Waals forces, π-cation bond, π-H bond and hydrogen bonding mechanisms. Also, by modifying the RF aerogel with graphene, the specific surface area increased, which could help adsorb more minocycline. Contact time of adsorbent and adsorbate, pH value, initial concentration and amount of adsorbent affected the amount of adsorption and removal percentage of minocycline antibiotic. Moreover, increasing the initial concentration had an adverse effect on this rate, which was explained by the empty adsorption sites and the chance of antibiotic molecules occupying them. The modified aerogel could be reused for 5 cycles. Employing the sol–gel technique, the aerogels were fashioned and then subjected to drying under ambient conditions. The integration of graphene sheets into the aerogel structure brought about an augmentation in adsorption capacity while maintaining the integrity of its porous architecture. In essence, graphene contributed to the enhancement of the specific surface area of the aerogel, thereby bolstering its proficiency in antibiotic absorption. At ideal pH values of 4 and 6, respectively, RF and RF-G1 aerogels removed 71.6 and 92.1% of minocycline. According to the IUPAC classification, the adsorption isotherms of RF and RF-G1 were type-IV adsorption isotherms. The RF and RF-G1 aerogels closely fitted kinetically with pseudo-second-order kinetics. According to the data obtained from the Langmuir model, they had a closer fitting among the fitted isotherms, indicating the limitation of the monolayer adsorption hypothesis in this study. Accordingly, the prepared aerogel could be a promising candidate for removing antibiotics from water, and therefore, have a cleaner environment.

## Data Availability

All data generated or analyzed during this study are included in this published article.

## References

[1] Krithiga T (2022). Persistent organic pollutants in water resources: Fate, occurrence, characterization and risk analysis. Sci. Total Environ..

[2] Liu Y (2022). Recent progress regarding electrochemical sensors for the detection of typical pollutants in water environments. Anal. Sci..

[3] Chang D, Mao Y, Qiu W, Wu Y, Cai B (2023). The source and distribution of tetracycline antibiotics in China: A review. Toxics.

[4] Maarouf L, Amin M, Evans BA, Abouelfetouh A (2023). Knowledge, attitudes and behaviour of Egyptians towards antibiotic use in the community: Can we do better?. Antimicrob. Resist. Infect. Control.

[5] Mutuku C, Gazdag Z, Melegh S (2022). Occurrence of antibiotics and bacterial resistance genes in wastewater: Resistance mechanisms and antimicrobial resistance control approaches. World J. Microbiol. Biotechnol..

[6] Serwecińska L (2020). Antimicrobials and antibiotic-resistant bacteria: A risk to the environment and to public health. Water.

[7] Zhao X-L (2021). Effects of environmental norfloxacin concentrations on the intestinal health and function of juvenile common carp and potential risk to humans. Environ. Pollut..

[8] Van Zyl KN, Matukane SR, Hamman BL, Whitelaw AC, Newton-Foot M (2022). Effect of antibiotics on the human microbiome: A systematic review. Int. J. Antimicrob. Agents.

[9] Liu X, Tang R, Li H, Wang L, Wan C (2022). The physiological and ecological properties of bacterial persisters discovered from municipal sewage sludge and the potential risk. Environ. Res..

[10] Liu Q (2023). Integrated photocatalysis and moving bed biofilm reactor (MBBR) for treating conventional and emerging organic pollutants from synthetic wastewater: Performances and microbial community responses. Bioresour. Technol..

[11] Li Z, Junaid M, Chen G, Wang J (2022). Interactions and associated resistance development mechanisms between microplastics, antibiotics and heavy metals in the aquaculture environment. Rev. Aquac..

[12] Mai Z (2023). Occurrence, distribution, and ecological risks of antibiotics in Honghu Lake and surrounding aquaculture ponds, China. Environ. Sci. Pollut. Res..

[13] Sodhi KK, Kumar M, Singh DK (2021). Insight into the amoxicillin resistance, ecotoxicity, and remediation strategies. J. Water Process Eng..

[14] Kumar S, Kumar S (2016). Miscellaneous bacteria. Essentials of Microbiology.

[15] Korajkic A, McMinn BR, Staley ZR, Ahmed W, Harwood VJ (2020). Antibiotic-resistant Enterococcus species in marine habitats: A review. Curr. Opin. Environ. Sci. Heal..

[16] Pattnaik A, Sahu JN, Poonia AK, Ghosh P (2023). Current perspective of nano-engineered metal oxide based photocatalysts in advanced oxidation processes for degradation of organic pollutants in wastewater. Chem. Eng. Res. Des..

[17] Kumar R (2022). A review on emerging water contaminants and the application of sustainable removal technologies. Case Stud. Chem. Environ. Eng..

[18] Ghafoori S (2022). New advancements, challenges, and future needs on treatment of oilfield produced water: A state-of-the-art review. Sep. Purif. Technol..

[19] Rekhate CV, Srivastava JK (2020). Recent advances in ozone-based advanced oxidation processes for treatment of wastewater—A review. Chem. Eng. J. Adv..

[20] Liu L (2021). Treatment of industrial dye wastewater and pharmaceutical residue wastewater by advanced oxidation processes and its combination with nanocatalysts: A review. J. Water Process Eng..

[21] Singh S (2021). Adsorption and detoxification of pharmaceutical compounds from wastewater using nanomaterials: A review on mechanism, kinetics, valorization and circular economy. J. Environ. Manag..

[22] Choi Y-K (2020). Adsorption behavior of tetracycline onto *Spirulina* sp.(microalgae)-derived biochars produced at different temperatures. Sci. Total Environ..

[23] Palacio DA, Becerra Y, Urbano BF, Rivas BL (2020). Antibiotics removal using a chitosan-based polyelectrolyte in conjunction with ultrafiltration membranes. Chemosphere.

[24] Xu L, Campos LC, Canales M, Ciric L (2020). Drinking water biofiltration: Behaviour of antibiotic resistance genes and the association with bacterial community. Water Res..

[25] Abbasnia A (2022). Removal of tetracycline antibiotics by adsorption and photocatalytic-degradation processes in aqueous solutions using metal organic frameworks (MOFs): A systematic review. Inorg. Chem. Commun..

[26] Wang T, Xue L, Liu Y, Zhang L, Xing B (2022). N self-doped hierarchically porous carbon derived from biomass as an efficient adsorbent for the removal of tetracycline antibiotics. Sci. Total Environ..

[27] Liu M (2013). MCM-41 impregnated with A zeolite precursor: Synthesis, characterization and tetracycline antibiotics removal from aqueous solution. Chem. Eng. J..

[28] Kumar Sarangi P (2023). Utilization of agricultural waste biomass and recycling toward circular bioeconomy. Environ. Sci. Pollut. Res..

[29] Paul NM, Harikumar VS (2022). Pyrolytic transformation of indigenous biomass wastes into biochar: An insight into char structure and physicochemical characteristics. J. Agric. Rural Dev. Trop. Subtrop..

[30] Jha S, Gaur R, Shahabuddin S, Tyagi I (2023). Biochar as sustainable alternative and green adsorbent for the remediation of noxious pollutants: A comprehensive review. Toxics.

[31] Fan S (2018). Removal of tetracycline from aqueous solution by biochar derived from rice straw. Environ. Sci. Pollut. Res..

[32] Amalina F (2022). Biochar production techniques utilizing biomass waste-derived materials and environmental applications–A review. J. Hazard. Mater. Adv..

[33] Picos-Corrales LA (2023). Chitosan as an outstanding polysaccharide improving health-commodities of humans and environmental protection. Polymers.

[34] Abd El-Monaem EM (2022). Sustainable adsorptive removal of antibiotic residues by chitosan composites: An insight into current developments and future recommendations. Arab. J. Chem..

[35] Jones CW (2022). Metal–organic frameworks and covalent organic frameworks: Emerging advances and applications. JACS Au.

[36] Zhang X, Peng F, Wang D (2022). MOFs and MOF-derived materials for antibacterial application. J. Funct. Biomater..

[37] Zhang Y, Liu Q, Yang C, Wu S, Cheng J (2019). Magnetic aluminum-based metal organic framework as a novel magnetic adsorbent for the effective removal of minocycline from aqueous solutions. Environ. Pollut..

[38] Hasanpour M, Hatami M (2020). Application of three dimensional porous aerogels as adsorbent for removal of heavy metal ions from water/wastewater: A review study. Adv. Colloid Interface Sci..

[39] Kéri M, Nagy B, László K, Bányai I (2021). Structural changes in resorcinol formaldehyde aerogel seen by NMR. Microporous Mesoporous Mater..

[40] Behzadi A, Yazdanbakhsh A (2022). Synthesis and characterization of modified resorcinol formaldehyde aerogel by graphene/m-phenylenediamine as a novel adsorbent to remove tetracycline antibiotics from wastewater. J. Water Environ. Nanotechnol..

[41] Rastegardoost MM (2023). Recent advances on porous materials and structures for high-performance triboelectric nanogenerators. Nano Energy.

[42] Li C (2023). Silica aerogels: From materials research to industrial applications. Int. Mater. Rev..

[43] Pinelli F, Piras C, Rossi F (2022). A perspective on graphene based aerogels and their environmental applications. FlatChem.

[44] Osman SH, Kamarudin SK, Basri S, Karim NA (2022). Organic aerogel as electro-catalytic support in low-temperature fuel cell. Int. J. Energy Res..

[45] Li L, Wang X, Li M (2022). A novel process to fabricate ultralight, intact mullite (3Al_2_O_3_·2SiO_2_) aerogel bulk. Mater. Lett..

[46] Guerrero-Alburquerque N (2020). Strong, machinable, and insulating chitosan-urea aerogels: Toward ambient pressure drying of biopolymer aerogel monoliths. ACS Appl. Mater. Interfaces.

[47] Bratovcic A, Petrinic I, Karabegović I (2020). Carbon based aerogels and xerogels for removing of toxic organic compounds. Lecture Notes in Networks and Systems.

[48] Nguyen VT (2023). Antibiotics tetracycline adsorption and flame-retardant capacity of eco-friendly aerogel-based nanocellulose, graphene oxide, polyvinyl alcohol, and sodium bicarbonate. J. Environ. Chem. Eng..

[49] Li T (2022). Construction of a novel highly porous BiOBr/CsxWO_3_@SiO_2_ composite aerogel: Adsorption/self-heating photocatalytic synergistic degradation of antibiotics and mechanism study. J. Environ. Chem. Eng..

[50] Li N, Tao K, Xia W, Yu C, Yang H (2023). A novel cellulose/lignin/montmorillonite ternary hybrid aerogel for efficiently adsorptive removal of antibiotics from water. Chem. Eng. J..

[51] Nasrollahi N, Vatanpour V, Khataee A (2022). Removal of antibiotics from wastewaters by membrane technology: Limitations, successes, and future improvements. Sci. Total Environ..

[52] Li M-F, Liu Y-G, Zeng G-M, Liu N, Liu S-B (2019). Graphene and graphene-based nanocomposites used for antibiotics removal in water treatment: A review. Chemosphere.

[53] Liu Q (2020). Polyaniline as interface layers promoting the in-situ growth of zeolite imidazole skeleton on regenerated cellulose aerogel for efficient removal of tetracycline. J. Colloid Interface Sci..

[54] Joshi P, Sharma OP, Ganguly SK, Srivastava M, Khatri OP (2022). Fruit waste-derived cellulose and graphene-based aerogels: Plausible adsorption pathways for fast and efficient removal of organic dyes. J. Colloid Interface Sci..

[55] Behzadi A, Hashemi Motlagh G, Raef M, Motahari S (2022). Rational design of in-situ-modified resorcinol formaldehyde aerogels for removing chlortetracycline antibiotics from aqueous solutions. Polym. Eng. Sci..

[56] Awadallah-f A, Al-Muhtaseb S (2020). Carbon nanoparticles-decorated carbon nanotubes. Sci. Rep..

[57] Chadha N, Sharma R, Saini P (2021). A new insight into the structural modulation of graphene oxide upon chemical reduction probed by Raman spectroscopy and X-ray diffraction. Carbon Lett..

[58] Rawat PS, Srivastava RC, Dixit G, Asokan K (2020). Structural, functional and magnetic ordering modifications in graphene oxide and graphite by 100 MeV gold ion irradiation. Vacuum.

[59] Ortiz-Martínez VM, Gómez-Coma L, Ortiz A, Ortiz I (2020). Overview on the use of surfactants for the preparation of porous carbon materials by the sol-gel method: Applications in energy systems. Rev. Chem. Eng..

[60] Liu M, Gao Y, Wang Y, Li Y, Zou D (2022). Status and opportunities in the treatment of tetracyclines from aquatic environments by metal-organic frameworks (MOFs) and MOFs-based composites. Mater. Today Chem..

[61] Ratnam MV (2023). Carbon-based nanoadsorbents for the removal of emerging pollutants. Adsorpt. Sci. Technol..

[62] Zhang H (2022). Performance and mechanism of sycamore flock based biochar in removing oxytetracycline hydrochloride. Bioresour. Technol..

[63] Guo S, Garaj S, Bianco A, Ménard-Moyon C (2022). Controlling covalent chemistry on graphene oxide. Nat. Rev. Phys..

[64] Hacıosmanoğlu GG (2022). Antibiotic adsorption by natural and modified clay minerals as designer adsorbents for wastewater treatment: A comprehensive review. J. Environ. Manag..

[65] Rayappa MK, Rattu GKS, Krishna PM (2023). Advances and effectiveness of metal–organic framework based bio/chemical sensors for rapid and ultrasensitive probing of antibiotic residues in foods Sustain. Food Technol..

[66] Dou Y, Bai Q, Guo W, Chen S, Wang H (2022). The influences of internal interactions and functional groups on the adsorption of graphene and graphene oxide with alumina substrate. Comput. Mater. Sci..

[67] Alatalo SM (2019). Mechanistic insight into efficient removal of tetracycline from water by Fe/graphene. Chem. Eng. J..

[68] Qi X, Jiang F, Zhou M, Zhang W, Jiang X (2021). Graphene oxide as a promising material in dentistry and tissue regeneration: A review. Smart Mater. Med..

[69] Zhao X (2023). Post-synthesis introduction of dual functional groups in metal–organic framework for enhanced adsorption of moxifloxacin antibiotic. J. Colloid Interface Sci..

[70] Srivastava A (2022). Low cost iron modified *syzygium*
*cumini* l. Wood biochar for adsorptive removal of ciprofloxacin and doxycycline antibiotics from aqueous solution. Inorg. Chem. Commun..

[71] Saghir S, Xiao Z (2021). Synthesis of novel Ag@ZIF-67 rhombic dodecahedron for enhanced adsorptive removal of antibiotic and organic dye. J. Mol. Liq..

[72] Padmavathy KS, Madhu G, Haseena PV (2016). A study on effects of pH, adsorbent dosage, time, initial concentration and adsorption isotherm study for the removal of hexavalent chromium (Cr (VI)) from wastewater by magnetite nanoparticles. Procedia Technol..

[73] Meena AK, Mishra GK, Rai PK, Rajagopal C, Nagar PN (2005). Removal of heavy metal ions from aqueous solutions using carbon aerogel as an adsorbent. J. Hazard. Mater..

[74] Saghir S, Xiao Z (2021). Facile preparation of metal-organic frameworks-8 (ZIF-8) and its simultaneous adsorption of tetracycline (TC) and minocycline (MC) from aqueous solutions. Mater. Res. Bull..

[75] Cavalcante EHM (2022). 3-Aminopropyl-triethoxysilane-functionalized tannin-rich grape biomass for the adsorption of methyl orange dye: Synthesis, characterization, and the adsorption mechanism. ACS Omega.

[76] dos Reis GS (2023). Preparation of highly porous nitrogen-doped biochar derived from birch tree wastes with superior dye removal performance. Colloids Surf. A Physicochem. Eng. Asp..

[77] dos Reis GS (2023). Synthesis of novel mesoporous selenium-doped biochar with high-performance sodium diclofenac and reactive orange 16 dye removals. Chem. Eng. Sci..

[78] Abin-Bazaine A, Campos Trujillo A, Olmos-Marquez M, Ince M, Ince OK (2022). Adsorption isotherms: Enlightenment of the phenomenon of adsorption. Wastewater Treatment.

[79] Brigante M, Schulz PC (2012). Cerium (IV) oxide: Synthesis in alkaline and acidic media, characterization and adsorption properties. Chem. Eng. J..

[80] Lu L, Li J, Yu J, Song P, Ng DHL (2016). A hierarchically porous MgFe_2_O_4_/γ-Fe_2_O_3_ magnetic microspheres for efficient removals of dye and pharmaceutical from water. Chem. Eng. J..

[81] Wang R (2021). Re-use of wasted sludge to treat industrial pollutants. Water Air Soil Pollut..

